# Mortality due to Vegetation Fire–Originated PM_2.5_ Exposure in Europe—Assessment for the Years 2005 and 2008

**DOI:** 10.1289/EHP194

**Published:** 2016-07-29

**Authors:** Virpi Kollanus, Marje Prank, Alexandra Gens, Joana Soares, Julius Vira, Jaakko Kukkonen, Mikhail Sofiev, Raimo O. Salonen, Timo Lanki

**Affiliations:** 1 Department of Health Protection, National Institute for Health and Welfare, Kuopio, Finland; 2 Atmospheric Composition Research, Finnish Meteorological Institute, Helsinki, Finland; 3 IER (Institute for Energy Economics and the Rational Use of Energy), University of Stuttgart, Stuttgart, Germany; 4 Unit of Public Health and Clinical Nutrition, University of Eastern Finland, Kuopio, Finland

## Abstract

**Background:**

Vegetation fires can release substantial quantities of fine particles (PM2.5), which are harmful to health. The fire smoke may be transported over long distances and can cause adverse health effects over wide areas.

**Objective:**

We aimed to assess annual mortality attributable to short-term exposures to vegetation fire–originated PM2.5 in different regions of Europe.

**Methods:**

PM2.5 emissions from vegetation fires in Europe in 2005 and 2008 were evaluated based on Moderate Resolution Imaging Spectroradiometer (MODIS) satellite data on fire radiative power. Atmospheric transport of the emissions was modeled using the System for Integrated modeLling of Atmospheric coMposition (SILAM) chemical transport model. Mortality impacts were estimated for 27 European countries based on a) modeled daily PM2.5 concentrations and b) population data, both presented in a 50 × 50 km2 spatial grid; c) an exposure–response function for short-term PM2.5 exposure and daily nonaccidental mortality; and d) country-level data for background mortality risk.

**Results:**

In the 27 countries overall, an estimated 1,483 and 1,080 premature deaths were attributable to the vegetation fire–originated PM2.5 in 2005 and 2008, respectively. Estimated impacts were highest in southern and eastern Europe. However, all countries were affected by fire-originated PM2.5, and even the lower concentrations in western and northern Europe contributed substantially (~ 30%) to the overall estimate of attributable mortality.

**Conclusions:**

Our assessment suggests that air pollution caused by PM2.5 released from vegetation fires is a notable risk factor for public health in Europe. Moreover, the risk can be expected to increase in the future as climate change proceeds. This factor should be taken into consideration when evaluating the overall health and socioeconomic impacts of these fires.

**Citation:**

Kollanus V, Prank M, Gens A, Soares J, Vira J, Kukkonen J, Sofiev M, Salonen RO, Lanki T. 2017. Mortality due to vegetation fire–originated PM2.5 exposure in Europe—assessment for the years 2005 and 2008. Environ Health Perspect 125:30–37; http://dx.doi.org/10.1289/EHP194

## Introduction

Vegetation fires, which include wildfires, prescribed forest fires, and open-field burnings related to agricultural practices, release large quantities of combustion-originated air pollutants (Naeher et al. 2007). Of these, particulate matter, and especially fine particles (PM_2.5_, particles with aerodynamic diameter < 2.5 μm), are considered the most harmful to public health. PM_2.5_ consists of a complex mixture of aerosols, part of which can remain suspended in the atmospheric surface layer for up to a week. PM_2.5_ infiltrates efficiently into buildings and enters the lower respiratory tract when inhaled. Numerous epidemiological studies have shown exposure to ambient PM_2.5_ to be associated with respiratory and cardiovascular morbidity and mortality (Pope and Dockery 2006).

Vegetation fires can episodically increase local and regional concentrations of airborne particles manyfold, even by orders of magnitude (Heil and Goldammer 2001; Liu et al. 2015; van Donkelaar et al. 2011). Daily averaged PM_2.5_ concentrations near areas affected by fire may reach several hundreds of micrograms per cubic meter, and pollution episodes can last from a few days to weeks. For comparison, the World Health Organization (WHO) health-based guideline value for daily average PM_2.5_ concentration is 25 μg/m^3^ (WHO 2006). However, the deterioration of air quality is not locally or even regionally limited. If the meteorological conditions are favorable, long-range transport of the fire-originated PM_2.5_ can result in significant increases in short-term exposure levels hundreds or even thousands of kilometers away from the fire-affected area (Niemi et al. 2009; Sapkota et al. 2005).

Globally, PM_2.5_ exposure from vegetation fires has been estimated to result in > 300,000 premature deaths annually (Johnston et al. 2012). Nearly 80% of these deaths occur in sub-Saharan Africa and Southeast Asia because the majority of the fire emissions originate from savannas and tropical forests (van der Werf et al. 2010), causing recurrent air pollution episodes in these densely populated regions. However, vegetation fire–originated air pollution has also been suggested to cause substantial public health impacts in developed countries (Hänninen et al. 2009; Kochi et al. 2012; Moeltner et al. 2013).

In Europe, the southernmost countries experience heavy impacts from vegetation fires. Between 2000 and 2013, 170,000 to 740,000 hectares of land overall were burned annually by uncontrolled forest, bush, and grassland fires in Portugal, Spain, Italy, Greece, and southern France [European Forest Fire Information System (EFFIS) 2015]. Air quality in Europe is also affected by fires in eastern European countries and in western Russia (Niemi et al. 2009; Witham and Manning 2007), where the common practice of using open-field fires in cropland management is a significant source of emissions (Korontzi et al. 2006; van der Werf et al. 2010). In the Black Sea region, agricultural burnings dominate vegetation-fire emissions (van der Werf et al. 2010). However, information about the public health significance of air pollution from vegetation fires in Europe is very limited. This study aimed to bridge this information gap by providing quantitative estimates for premature mortality among the general population caused by short-term exposures to fire-originated PM_2.5_ in different European regions.

## Materials and Methods

### Emissions and Atmospheric Transport of Vegetation Fire–Originated PM_2.5_

PM_2.5_ emissions from vegetation fires for the years 2005 and 2008 were computed using the Integrated Monitoring and Modelling System for Wildland Fires (IS4FIRES) [Finnish Meteorological Institute (FMI) 2016; Sofiev et al. 2009]. The system utilizes the fire radiative power (FRP) (Kaufman et al. 1998) observations of the Moderate Resolution Imaging Spectroradiometer (MODIS) instruments onboard the Aqua and Terra research satellites of the United States National Aeronautics and Space Administration (NASA). FRP is assumed to be proportional to the biomass burning rate, which is converted to PM_2.5_ emission using land-use dependent empirical emission factors of IS4FIRESv2 (Soares et al. 2015). The Global Land Cover Characterization (GLCC) land-use inventory (Loveland et al. 2000), combined with seven vegetation classes described in Akagi et al. (2011), was used to describe the spatial distribution of different land covers.

Atmospheric transport of the vegetation fire–originated PM_2.5_ was modeled using the System for Integrated modeLling of Atmospheric coMposition (SILAM) chemical transport model (http://silam.fmi.fi; Sofiev et al. 2015). The computations were based on meteorological data (temperature, pressure, wind, humidity, precipitation, cloud cover, and solar radiation) from operational forecasts of the European Centre for Medium-Range Weather Forecasts (ECMWF 2016).

**Figure 1:**
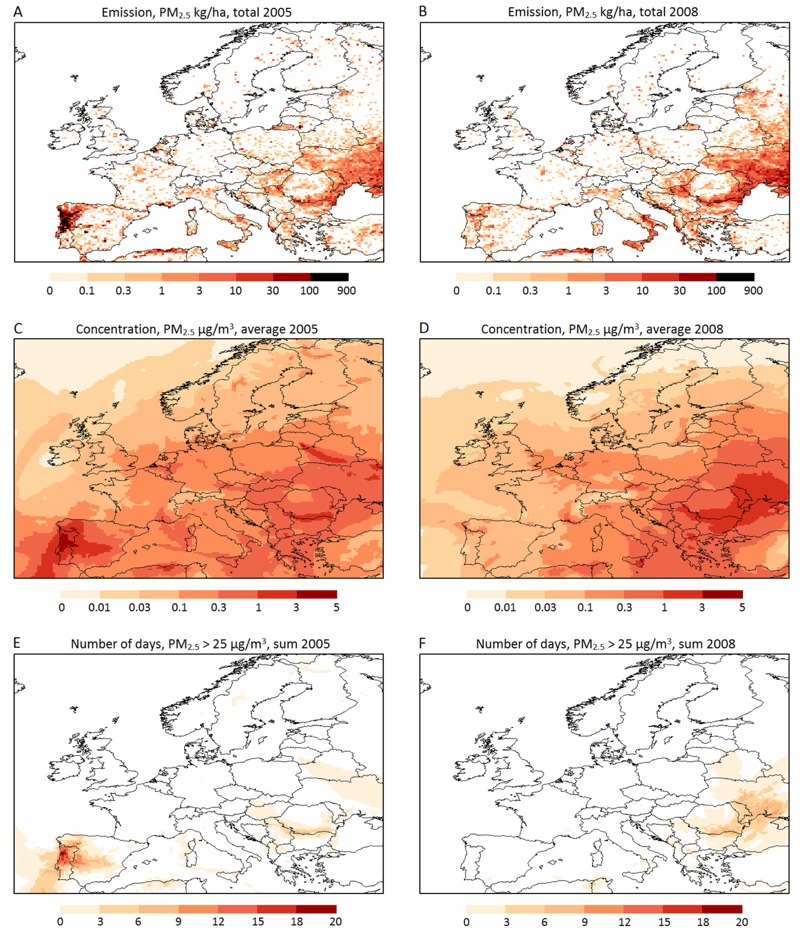
Modeled emissions and atmospheric transport of vegetation fire–originated fine particles (PM_2.5_) for 2005 and 2008. (*A*,*B*) annual emissions (kilograms/hectare); (*C,D*) annual average concentrations (micrograms/cubic meter); and (*E,F*) the number of days when the daily average concentration of fire-originated PM_2.5_ exceeded the WHO health-based guideline value of 25 μg/m^3^. Left panels represent year 2005, right panels represent year 2008. Emissions were modeled using the Integrated Monitoring System for Wildland Fires (IS4FIRES; FMI 2016). Atmospheric transport was modeled using the System for Integrated modeLling of Atmospheric coMposition (SILAM; http://silam.fmi.fi) chemical transport model.

The emission and atmospheric transport computations covered all of Europe (35°–70° N, 15° W–35° E, see Figure 1) with spatial resolution of 0.3° × 0.2°, temporal resolution of 15 min and vertical grids consisting of eight unevenly spaced layers stacked up to a height of ~8 km. Plume rise was estimated for every fire at every model time step by the algorithm of Sofiev et al. (2012).

To match with data for population distribution, the results of the fire-originated PM_2.5_ simulations—maps of aerosol concentrations—were interpolated to the European Monitoring and Evaluation Programme (EMEP) standard grid (spatial resolution 50 × 50 km^2^; EMEP 2016).

The years 2005 and 2008 were selected for this analysis because they coincide with relevant data sets available from other projects. In particular, an evaluation of the performance of the SILAM transport model is available for 2005 (Prank et al. 2016), and detailed calibration of the IS4FIRES fire emission data is available for 2008 (Soares et al. 2015).

### Spatial Distribution of Population

To assess the number and spatial distribution of the exposed population, we used a population data set developed in the European Union (EU)-funded Integrated Assessment of Health Risk of Environmental Stressors in Europe (INTARESE) project (Briggs 2008). The data set provides spatially distributed, age- and sex-stratified data for the current and future European population (EU-27, Iceland, Norway, and Switzerland) in the EMEP standard grid (50 × 50 km^2^). In the present assessment, we used gridded estimates for total population in 2010 because it was the nearest available year among the years for which air pollution from vegetation fires were modeled (2005 and 2008).

The population data set and a description of its development are available in the Integrated Environmental Health Impact Assessment System (IEHIAS 2011). Briefly, the data set was developed on the basis of *a*) census data from 23 European countries stratified by age and sex and available on the European Local Administrative Unit (LAU) level 2 (municipalities or equivalent units) (IEHIAS 2010), *b*) United Nations (UN) population data stratified by age and sex (UN 2016), and *c*) Gridded World Population (GWP) data on the spatial distribution of the population [Socioeconomic Data and Applications Center (SEDAC) 2016]. The spatial distribution of the population (age- and sex-stratified) in Europe was determined for the year 2000 by intersecting the census data (in 5-year age groups and stratified by sex) for LAU 2 regions with EMEP grid cells. If country census data were not available for 5-year age groups, the population was allocated to 5-year age groups based on the age distribution of the country’s population in the UN data. For countries without census data, the spatial distribution of the population was based on the age- and sex-stratified UN country population estimates that were area-weighted based on the GWP data. Finally, population growth rates from the UN data (for each country and population subgroup separately) were used to project the data from 2000 to the future years.

### Mortality Impact Analysis

Within all countries included in our mortality assessment, only 4–5% of the modeled vegetation fire–originated daily average grid-cell PM_2.5_ concentrations in 2005 and 2008 were > 1 μg/m^3^, and < 1% were > 5 μg/m^3^ (see Tables S1 and S2). Therefore, exposure to moderate or high levels of vegetation-fire smoke was sporadic, and mortality due to the smoke exposure was evaluated on the basis of acute mortality risk related to short-term increases in PM_2.5_ concentrations. First, the relative mortality risk caused by the fire-originated PM_2.5_ in each grid-cell and day of the year was calculated as

RR´ = exp(ln(RR)/10 × *C*), [1]

where RR is the relative-risk exposure–response function for daily PM_2.5_ exposure and nonaccidental mortality, and *C* is the modeled daily average concentration of the fire-originated PM_2.5_ (micrograms/cubic meter) in the grid-cell. For the exposure–response function, we assumed an RR of 1.0098 per 10 μg/m^3^ [95% confidence interval (CI): 1.0075, 1.0122], that is, 0.98% increase in the risk of nonaccidental mortality per each 10 μg/m^3^ increment in the source-specific PM_2.5_ concentration. The estimate is based on an epidemiological study on the mortality effects of short-term urban PM_2.5_ exposures in 112 U.S. cities (Zanobetti and Schwartz 2009).

Next, the population attributable fraction (PAF), that is, the fraction of population mortality attributable to the fire-originated PM_2.5_, was calculated for each grid-cell and day as

*PAF* = (RR´ – 1)/RR´. [2]

Finally, the number of daily deaths attributable to the fire-originated PM_2.5_ in each grid-cell was calculated as

Attributable deaths = P × *MR* × *PAF*, [3]

where P is the country-specific population in the grid-cell, and MR is the country-specific daily background mortality risk. For each country, the daily background risk for nonaccidental mortality in 2005 and 2008 was estimated on the basis of the yearly mortality statistics from the World Health Organization (WHO) Mortality Database (WHO 2013c). If country data were not available for these years, data for the nearest possible year were used. The daily average mortality risk was defined by dividing the annual mortality risk by the number of days in the year (365 in 2005, 366 in 2008). Finally, the daily attributable deaths were summed over all days of the year and country- or region-specific grid-cells.

We assumed a linear exposure–response function at all vegetation fire–originated PM_2.5_ concentration levels. However, the applied exposure–response function is valid for short-term urban levels of PM_2.5_ commonly encountered in developed countries (daily concentrations generally well below 100 μg/m^3^). Because there were some higher values among the modeled fire-originated daily PM_2.5_ concentrations, and because the cardiovascular mortality effects of long-term exposures have been suggested to flatten at high PM levels (Pope et al. 2011), we wanted to test the sensitivity of the assessment outcome to the possible overestimation of the mortality effect at high vegetation fire–originated PM_2.5_ levels. To this end, we performed an additional analysis in which the mortality impacts were calculated by fixing the modeled daily PM_2.5_ concentrations exceeding 100 μg/m^3^ to be equal to 100 μg/m^3^.

The mortality impacts associated with vegetation fire–originated PM_2.5_ were assessed for all 27 European countries for which all required input data were available. The countries were further classified into northern, eastern, western, and southern European regions (see Table 1).

**Table 1:** Population-weighted annual average concentration*^a^* (micrograms/cubic meter) of vegetation fire–originated fine particles (PM_2.5_).

Region, country	2005	2008
Northern Europe	0.07	0.04
Denmark	0.10	0.05
Finland	0.06	0.05
Norway	0.04	0.02
Sweden	0.08	0.04
Eastern Europe	0.39	0.45
Bulgaria	0.67	1.11
Czech Republic	0.26	0.15
Estonia	0.09	0.11
Hungary	0.48	0.39
Latvia	0.12	0.13
Lithuania	0.25	0.17
Poland	0.25	0.16
Romania	0.66	1.08
Slovenia	0.21	0.15
Slovakia	0.39	0.26
Western Europe	0.19	0.12
Austria	0.28	0.23
Belgium	0.33	0.20
France	0.17	0.10
Germany	0.24	0.16
Ireland	0.02	0.03
Luxembourg	0.35	0.13
Netherlands	0.27	0.18
Switzerland	0.08	0.05
United Kingdom	0.09	0.08
Southern Europe	0.57	0.26
Greece	0.41	0.79
Italy	0.25	0.33
Portugal	2.77	0.06
Spain	0.52	0.08
All regions	0.32	0.22
^***a***^Estimated based on emissions from the Integrated Monitoring and Modelling System for Wildland Fires (IS4FIRES; FMI 2016), the System for Integrated modeLling of Atmospheric coMposition (SILAM; http://silam.fmi.fi) chemical transport model, and INTARESE data on population distribution in Europe (IEHIAS 2011).		

## Results

### Emissions and Atmospheric Transport of Vegetation Fire–Originated PM_2.5_

The large-scale distribution of the modeled vegetation-fire emissions in 2005 and 2008 were similar: the fires were intensive mainly in northern Portugal and Spain, southern Italy, the Balkans, the Black Sea area, the Kaliningrad area, and other parts of western Russia (Figure 1A,B; see also Table S3 for country-specific emission estimates). The main difference between the 2 years was in the strength of the fires in the southern and eastern regions, which can be explained, at least in part, by the differences in the fire-season (April–October) temperatures and in precipitation between 2005 and 2008 in these regions (see Figure S1). The conditions in the Iberian Peninsula in 2005 were hot and dry, leading to a large number of vegetation fires, whereas the cooler and wetter conditions in 2008 led to relatively few fires. In contrast, the Balkan region and southern Italy were hotter and drier than average in 2008, resulting in more extensive fires than in 2005.

Model-based estimates of vegetation fire–originated PM_2.5_ concentrations (Figure 1C,D) follow the spatial distributions for the vegetation-fire emissions (Figure 1A,B), but the smoke affected wider regions than the fire areas. In 2005, the highest concentrations were located in northern Portugal and Spain, whereas in 2008, the pollution was highest in countries bordering the Black Sea. In northern Europe, winds during the fire season (April–October) were stronger and more frequently northerly in 2005 than in 2008 (see Figure S2). As a consequence, long-range transported vegetation-fire smoke reached higher latitudes in 2005 than in 2008. However, long-term mean statistics of wind patterns do not adequately characterize yearly variations in population smoke exposure, which depend strongly on the short-term wind directions and other conditions affecting smoke aerosol transport during the fire events.

The WHO health-based guideline value for daily PM_2.5_ (25 μg/m^3^) was exceeded solely due to the vegetation fire–originated smoke only in a few places and on a few days (Figure 1E,F). However, in Portugal, the exceedance lasted for approximately 3 weeks in 2005.

Population-weighted annual average concentrations of the vegetation fire–originated PM_2.5_ in different countries ranged from 0.02 to 2.8 μg/m^3^ in 2005 and from 0.02 to 1.1 μg/m^3^ in 2008 (Table 1). Between 2005 and 2008, there were substantial country-level variations in exposure, particularly within the southern and eastern regions of Europe. However, in both 2005 and 2008, the regional-level population-weighted concentrations were clearly highest in the south and the east and lowest in the north.

### Mortality Impact

**Figure 2:**
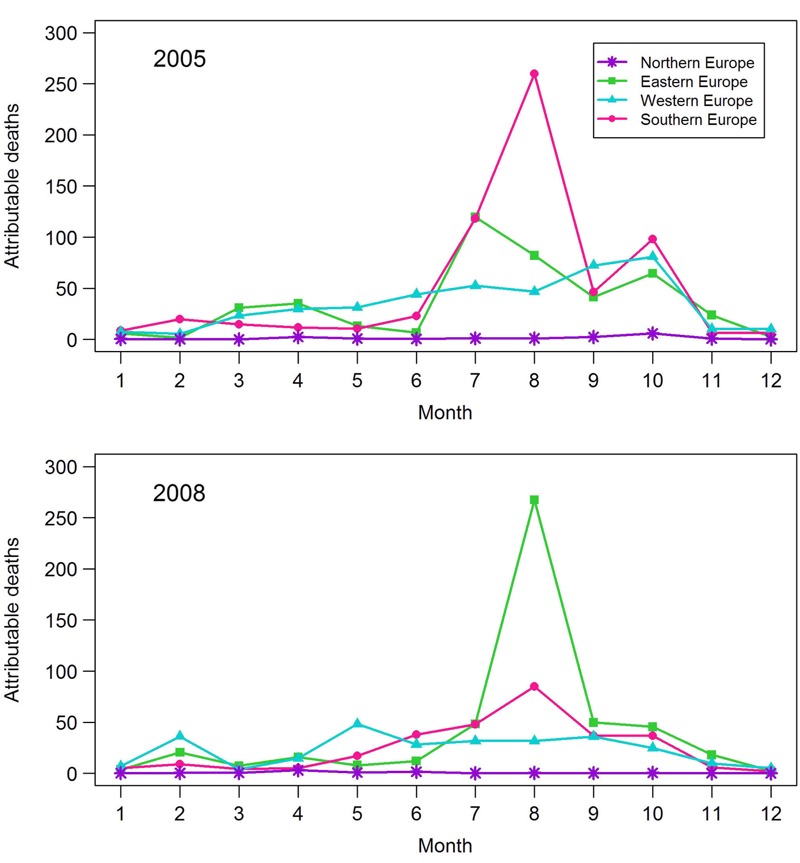
Monthly distribution of premature deaths attributable to vegetation fire–originated fine particles (PM_2.5_) in different regions of Europe. Numbers represent months beginning from January. Attributable mortality was estimated based on a relative risk of 1.0098 per 10 μg PM_2.5_/m^3^ (Zanobetti and Schwartz 2009), INTARESE data on population distribution in Europe (IEHIAS 2011), and background mortality data from the WHO Mortality Database (WHO 2013c). For material drawn from the World Health Organization (WHO) Mortality Database, analyses, interpretations, or conclusions are those of the authors and not of WHO, which is responsible only for the provision of the original information.

In the 27 countries assessed, we estimated that 1,483 and 1,080 premature deaths were attributable to vegetation fire–originated PM_2.5_ in 2005 and 2008, respectively, assuming RR = 1.0098 for a 10 μg/m^3^ increase in PM_2.5_ (Table 2). Ranges of uncertainty estimated using the lower and upper 95% confidence interval bounds for the exposure–response function from Zanobetti and Schwartz (2009) (RR = 1.0075 and 1.0122, respectively) were 1,139–1,839 for attributable premature deaths in 2005, and 828–1,342 premature deaths in 2008. In absolute terms, the estimated numbers of deaths attributable to vegetation fire–originated PM_2.5_ were comparable in the southern, eastern, and western regions of Europe. In relative terms (per 100,000 inhabitants), the attributable mortality was clearly highest in the southern and eastern regions. In the south and the east, the mortality impacts peaked in summer, whereas in the north and the west, the monthly variation was less pronounced (Figure 2).

**Table 2:** Central estimate and uncertainty range for premature deaths*^a^* (total number and death rate per 100,000 inhabitants) attributable to vegetation fire–originated fine particles (PM_2.5_).

Region, country	Attributable deaths, total		Attributable deaths per 100,000	
	2005	2008	2005	2008
Northern Europe	17 (13, 21)	9 (7, 12)	0.07 (0.05, 0.08)	0.04 (0.03, 0.05)
Denmark	5 (4, 6)	3 (2, 3)	0.09 (0.07, 0.11)	0.05 (0.04, 0.06)
Finland	3 (2, 3)	2 (2, 3)	0.05 (0.04, 0.06)	0.04 (0.03, 0.05)
Norway	2 (1, 2)	1 (1, 1)	0.04 (0.03, 0.05)	0.02 (0.01, 0.02)
Sweden	7 (5, 9)	4 (3, 5)	0.08 (0.06, 0.09)	0.04 (0.03, 0.05)
Eastern Europe	428 (328, 531)	499 (383, 620)	0.42 (0.32, 0.52)	0.49 (0.38, 0.61)
Bulgaria	68 (52, 85)	112 (86, 139)	0.91 (0.70, 1.13)	1.50 (1.15, 1.86)
Czech Republic	26 (20, 32)	15 (11, 18)	0.25 (0.19, 0.31)	0.14 (0.11, 0.17)
Estonia	1 (1, 2)	2 (1, 2)	0.10 (0.08, 0.13)	0.12 (0.09, 0.15)
Hungary	58 (45, 72)	46 (35, 57)	0.59 (0.45, 0.73)	0.46 (0.35, 0.57)
Latvia	3 (3, 4)	4 (3, 4)	0.15 (0.12, 0.19)	0.16 (0.12, 0.20)
Lithuania	8 (7, 10)	6 (5, 8)	0.26 (0.20, 0.32)	0.19 (0.15, 0.24)
Poland	84 (64, 104)	54 (42, 67)	0.22 (0.17, 0.27)	0.14 (0.11, 0.18)
Romania	156 (119, 193)	246 (189, 306)	0.73 (0.56, 0.91)	1.16 (0.89, 1.44)
Slovenia	4 (3, 4)	2 (2, 3)	0.18 (0.13, 0.22)	0.12 (0.09, 0.15)
Slovakia	19 (15, 24)	13 (10, 16)	0.35 (0.27, 0.44)	0.23 (0.18, 0.29)
Western Europe	415 (318, 515)	278 (213, 345)	0.16 (0.12, 0.20)	0.11 (0.08, 0.14)
Austria	20 (15, 24)	16 (12, 20)	0.23 (0.18, 0.29)	0.19 (0.14, 0.23)
Belgium	32 (25, 40)	19 (14, 23)	0.30 (0.23, 0.37)	0.17 (0.13, 0.22)
France	83 (64, 103)	48 (36, 59)	0.13 (0.10, 0.17)	0.08 (0.06, 0.10)
Germany	186 (142, 231)	127 (97, 158)	0.22 (0.17, 0.28)	0.15 (0.12, 0.19)
Ireland	1 (1, 1)	1 (1, 1)	0.01 (0.01, 0.02)	0.02 (0.01, 0.02)
Luxembourg	1 (1, 2)	0 (0, 1)	0.25 (0.19, 0.31)	0.09 (0.07, 0.11)
Netherlands	35 (27, 43)	23 (18, 29)	0.21 (0.16, 0.26)	0.14 (0.11, 0.17)
Switzerland	4 (3, 5)	3 (2, 4)	0.06 (0.04, 0.07)	0.04 (0.03, 0.05)
United Kingdom	52 (40, 65)	42 (32, 52)	0.08 (0.06, 0.11)	0.07 (0.05, 0.08)
Southern Europe	624 (480, 772)	294 (225, 365)	0.50 (0.38, 0.61)	0.23 (0.18, 0.29)
Greece	40 (31, 50)	80 (62, 100)	0.36 (0.27, 0.45)	0.72 (0.55, 0.89)
Italy	133 (102, 166)	182 (139, 226)	0.22 (0.17, 0.28)	0.30 (0.23, 0.38)
Portugal	260 (201, 321)	5 (4, 7)	2.51 (1.94, 3.10)	0.05 (0.04, 0.06)
Spain	190 (146, 235)	26 (20, 33)	0.43 (0.33, 0.53)	0.06 (0.05, 0.07)
All regions	1,483 (1,139, 1,839)	1,080 (828, 1,342)	0.29 (0.22, 0.36)	0.21 (0.16, 0.26)
^***a***^Estimated based on a relative mortality risk of 1.0098 per 10 μg PM_2.5_/m^3^ (Zanobetti and Schwartz 2009), INTARESE data on population distribution in Europe (IEHIAS 2011), and background mortality data from the WHO Mortality Database (WHO 2013c). Uncertainty range is based on the upper and lower bounds of the 95% confidence interval (CI) of the exposure–response function (1.0075, 1.0122; Zanobetti and Schwartz 2009). For material drawn from the World Health Organization (WHO) Mortality Database, analyses, interpretations, or conclusions are those of the authors and not of WHO, which is responsible only for the provision of the original information.				

Overall, our estimates suggest that the majority (70–80%) of the premature deaths attributable to vegetation fire–originated PM_2.5_ in 2005 and 2008 were caused by relatively low to moderate increases in daily concentrations of PM_2.5_ (≤ 15 μg/m^3^). This phenomenon was seen particularly in the western and northern regions, where PM_2.5_ increases of ≤ 2 μg/m^3^ and ≤ 15 μg/m^3^ were estimated to have caused 60–80% and ~99% of the premature deaths in these regions, respectively (Table 3).

**Table 3:** Relative frequency of modeled daily average grid-cell concentrations*^a^* of vegetation fire–originated fine particles (PM_2.5_), and premature deaths*^b^* attributable to these concentrations.

Year, region	PM_2.5_ < 0.5 μg/m^3^		PM_2.5_ 0.5–2 μg/m^3^		PM_2.5_ 2–15 μg/m^3^		PM_2.5_ > 15 μg/m^3^	
	Relative frequency (%)	Attributable deaths (*n*)	Relative frequency (%)	Attributable deaths (*n*)	Relative frequency (%)	Attributable deaths (*n*)	Relative frequency (%)	Attributable deaths (*n*)
2005
Northern Europe	97.0	5	2.7	8	0.3	3	0.01	0.2
Eastern Europe	85.7	49	10.3	119	3.6	160	0.3	100
Western Europe	92.6	90	6.1	166	1.3	155	0.01	4
Southern Europe	88.1	54	7.8	82	3.5	167	0.7	320
All regions	90.8	198	6.7	375	2.2	485	0.3	424
2008
Northern Europe	98.8	4	1.0	3	0.2	2	0.001	0.1
Eastern Europe	87.8	54	7.7	87	3.9	202	0.6	157
Western Europe	95.2	80	4.1	119	0.7	77	0.004	1
Southern Europe	89.4	46	7.0	84	3.5	137	0.1	26
All regions	92.7	185	5.0	294	2.1	418	0.2	184
^***a***^Estimated based on emissions from the Integrated Monitoring System for Wildland Fires (IS4FIRES; FMI 2016) and the System for Integrated modeLling of Atmospheric coMposition (SILAM; http://silam.fmi.fi) chemical transport model. ^***b***^Estimated based on a relative mortality risk of 1.0098 per 10 μg PM_2.5_/m^3^ (Zanobetti and Schwartz 2009), INTARESE data on population distribution in Europe (IEHIAS 2011), and background mortality data from the WHO Mortality Database (WHO 2013c).								

The sensitivity analysis regarding the possible overestimation of the PM_2.5_ mortality effect at high exposure levels showed a negligible effect on the assessment outcome (data not shown): limiting the modeled vegetation fire–originated PM_2.5_ concentrations above 100 μg/m^3^ to 100 μg/m^3^ reduced the total mortality estimates for Europe by 1.6% and 0.01% for 2005 and 2008, respectively.

## Discussion

Wildfires can be catastrophic events leading to immediate losses of life and resulting in damage costs on the order of billions of Euro (San-Miguel-Ayanz et al. 2013). Although wildfires cause a large amount of public concern, their far-reaching effects on air quality are often ignored. Our assessment suggests that fine particles emitted from vegetation fires may have substantial effects on health throughout Europe, causing tens to hundreds of premature deaths in many countries each year. Hence, our estimates indicate that the overall mortality attributable to these fires is far higher and more widespread than the immediate fatalities reported from the fire-affected areas.

In 2005 and 2008, the modeled exposure to the vegetation fire–originated PM_2.5_ and the resulting mortality effect estimates were, on a regional level, several times higher in the fire-prone southern and eastern European countries than in the western and northern countries. In the south and the east, the estimated pollution and mortality impacts clearly peaked in July–August, which is the strongest fire season for both wildfires and agricultural-waste burnings in these areas (Korontzi et al. 2006; Magi et al. 2012). However, our findings suggest that the western and northern European countries were also affected, largely as a result of long-range transport of the fire smoke. In the west and the north, estimated annual fire-originated PM_2.5_ concentrations were, in general, clearly lower than those in the south and the east and were more evenly distributed throughout the spring, summer, and autumn months. However, exposures to these lower source-specific PM_2.5_ levels accounted for a substantial proportion of the estimated overall mortality impact of the vegetation-fire emissions in Europe because large numbers of people were exposed over extensive areas, particularly in western Europe.

In the 27 countries overall, we estimated that daily average fire-originated PM_2.5_ concentrations of ≤ 15 μg/m^3^, which are low to moderate compared with typical urban PM_2.5_ concentrations in Europe, accounted for > 70% of the premature deaths attributed to PM_2.5_ from vegetation fires. In the northern and western regions, we estimated that 60–80% of the attributable deaths resulted from increments < 2 μg/m^3^, which are small compared with normal daily variation in PM_2.5_ in European cities (up to tens of micrograms/cubic meter). However, our estimates also demonstrate the potential extent of health impacts arising from severe pollution episodes in large-scale fire events, such as the 2005 event in northwest Portugal (San-Miguel-Ayanz et al. 2013). During this type of event, PM_2.5_ levels in nearby areas can increase manyfold, leading to severe health effects over a period of several weeks. Furthermore, in areas with relatively low long-term levels of air pollution, increases in daily PM_2.5_ classified herein as moderate can, from a local perspective, mean substantial worsening of the air quality.

Although our assessment suggests widespread health effects of vegetation fire–originated PM_2.5_ in Europe, our exposure estimates did not account for fire PM_2.5_ transported to Europe from areas outside the emission grid used in our analysis (e.g., from central Asia and central Russia). More importantly, our mortality estimates did not include Ukraine, Belarus, and southwestern European Russia, which are hotspots for vegetation-fire emissions. Furthermore, the health effects of fire-originated PM_2.5_ are not limited to increases in mortality but include a wide range of less severe health outcomes, such as worsening of respiratory and cardiovascular diseases (Pope and Dockery 2006). In addition to PM_2.5_, fire smoke also contains other pollutants that are harmful to health, such as coarse thoracic particles (size-range 2.5–10 μm), nitrogen dioxide, ozone, and a wide variety of gaseous hydrocarbons (Naeher et al. 2007). Adverse health effects associated with the abovementioned factors were not considered in the present assessment.

Assessing only 2 years is, of course, a major limitation for drawing general conclusions on the health effects of vegetation fire–originated PM_2.5_ in Europe and raises questions regarding whether our exposure and mortality estimates represent the low or high end of the interannual variation in impacts. Owing to weather conditions, vegetation characteristics, and human factors (Flannigan et al. 2009), there are considerable yearly differences in fire activity, which was also manifested in our study. According to data from EFFIS, the area burned in the Iberian Peninsula in 2005 was the second largest, and in 2008 was the smallest, recorded from 2000 to 2013 (EFFIS 2015). The difference in the extent and severity of the fires between 2005 and 2008 was particularly striking in Portugal. Hence, the mortality estimates for the Iberian Peninsula, and particularly for Portugal, likely indicate the potential range of annual impacts in the 2000s.

Based on the IS4FIRES inventory for the whole of Europe, the overall emissions of vegetation fire–originated PM in 2005 and 2008 were not drastically different from those in other years between 2000 and 2013 (see Figure S3). This observation suggests that, apart from the Iberian Peninsula, our mortality estimates are unlikely to represent years with uncommonly high or low overall impacts in Europe. This idea is also supported by Giglio et al. (2013), who have provided burned-area estimates for different world regions in the 2000s based on the fourth-generation Global Fire Emissions Database (GFED4) inventory. However, exposures and health impacts of air pollution from vegetation fires are determined not only by the area burned or the total emissions but also depend on the proximity of fires to densely populated areas and the prevailing emission transport conditions during the fires. Therefore, the extent to which the regional mortality impacts in 2005 and 2008 can be generalized to other years remains unclear.

The validity of any health impact assessment on vegetation fire–originated air pollution depends on the quality of the emission and atmospheric transport modeling. In the IS4FIRES emission estimates used in the present assessment, there are uncertainties resulting from *a*) the strong diurnal variations in fire intensity that are not resolved by the MODIS FRP observations; *b*) difficulties in observing small fires, which occur quite frequently in Europe (leading to ~10% loss in emissions); and *c*) very limited information on burning characteristics and, consequently, emission factors for individual fires (Soares et al. 2015). Within the IS4FIRES estimates, these (potentially large) uncertainties are constrained during the system calibration step, which adjusts the emission factors based on a comparison of MODIS aerosol optical depth (AOD) observations of atmospheric PM and SILAM simulations of the fire-smoke distribution. In the AOD observations, it is not possible to distinguish the fire-originated PM from that originating from other sources. Therefore, the IS4FIRES system calibration is based on episodes with strongly dominant fire-induced pollution. However, such episodes are not common in Europe, where the contribution of fire to total PM is typically relatively small, thus increasing the uncertainty in the European fire emissions and making it difficult to assess their accuracy.

With regard to the atmospheric transport modeling, the main uncertainties include *a*) vertical distribution of the emissions, *b*) particle properties, and *c*) formation of secondary particles when fire-originated gaseous compounds released to the atmosphere participate in chemical reactions. However, calibration of the IS4FIRES vegetation-fire emission factors is based on observed AOD, with the majority of the fire plumes being at least several hours old. As a result, the majority of the secondary particle formation would have already occurred and is, therefore, included in the modeled fire-originated PM_2.5_ concentrations. Overall, when SILAM simulations of atmospheric PM_2.5_ total concentrations are compared with daily air quality observations from the EMEP observational network, SILAM performs reasonably well (Prank et al. 2016). As is the case with other regional transport models, SILAM tends to underestimate the observed PM levels mainly because some PM components, such as wind-blown dust, secondary organic aerosol, and aerosol-bound water, are omitted from the simulations. However, when compared with three other transport models, SILAM showed the smallest underestimation for total PM_2.5_ (~ 25%; Prank et al. 2016).

Nevertheless, our modeled concentrations of vegetation fire–originated PM_2.5_ are likely to overestimate the influence of strong fires. An indication for this overestimation is that when SILAM simulations of total PM_2.5_ are compared with PM_2.5_ measurements at a monitoring station affected by intensive fire episodes in summer months, the underestimation due to the PM components omitted from the simulations that is expected in the modeled total PM_2.5_ concentrations is not evident (see Figure S4A). Because the omitted components (wind-blown dust and secondary organic aerosol) are abundant during the summer, a negative bias between the modeled and measured PM_2.5_ is expected to remain during summer. This negative bias is, in fact, evident in a monitoring station not influenced by vegetation fires (see Figure S4B). Thus, it is likely that overestimation of PM_2.5_ from strong vegetation fires partly masks the underestimation due to the PM components missing from the simulations.

In contrast, because of difficulties in detecting small vegetation fires (Soares et al. 2015), PM_2.5_ emissions and exposure from small fires are likely underestimated in our assessment. Although the majority of wildfires are started by people and, therefore, occur near populated areas (Ganteaume et al. 2013), fires in inhabited regions are usually small in size compared with those occurring in remote areas (Archibald et al. 2009; Knorr et al. 2014). This is likely a result of the more fragmented landscape in inhabited areas, which limits the fire spread, and to more efficient fire suppression. However, because of the shorter distance to the population, and because smoke plumes from small fires stay near the ground, the impact of small fires on population exposure to PM_2.5_ can be substantial.

The accuracy of the mortality estimates also depends on whether the exposure–response function for urban PM_2.5_ is valid for estimating impacts of vegetation-fire PM_2.5_. Although the adverse effects of urban PM_2.5_ on respiratory and cardiovascular morbidity and mortality have been clearly demonstrated (Pope and Dockery 2006), epidemiological evidence of the effects of PM_2.5_ from biomass burning is scarcer. However, the present consensus based on the physical and chemical properties of the particles and on toxicological and epidemiological studies is that PM released from biomass burning should not be considered less harmful to human health than ambient air PM in general (Naeher et al. 2007; WHO 2013b). Epidemiological studies have found associations between exposure to wildfire smoke and respiratory health effects, and the evidence of the cardiovascular effects of such exposure has strengthened (Faustini et al. 2015; Liu et al. 2015; WHO 2013b; Youssouf et al. 2014). Associations with increased mortality have also been reported (Analitis et al. 2012; Faustini et al. 2015; Johnston et al. 2011; Sastry 2002), but few studies have attempted to quantify the size of the mortality effect per unit increase in vegetation-fire PM exposure.

Adverse health effects of air pollution may also vary between populations because of differences in, for example, exposure to outdoor air pollutants, population age structure, and prevalence of disease. Therefore, the exposure–response function used in our analysis, which was estimated for the U.S. population (Zanobetti and Schwartz 2009), may not be valid for estimating mortality impacts in different regions of Europe. Evidence of the short-term mortality effects of urban PM_2.5_ in Europe is less comprehensive than that in the United States. However, a meta-analysis of the existing European single- and multi-city studies suggests that the mortality effect of PM_2.5_ is similar in Europe (RR = 1.0123 per 10 μg/m^3^) (Héroux et al. 2015; WHO 2013a) and the United States (RR = 1.0098 per 10 μg/m^3^) (Zanobetti and Schwartz 2009).

Because of climate change, vegetation fires are a growing concern for the future (Flannigan et al. 2009). In Europe, the risk of wildfires has been projected to increase in most regions. Recent estimates for the potential increase in overall area burned by the end of the 21st century vary from 40% to 200% compared with the present situation (Khabarov et al. 2016; Migliavacca et al. 2013). The absolute increase in burned area is expected to be particularly high in southern and eastern Europe. However, in relative terms, the impacts may also be notable in central and northern Europe. Moreover, because fires often coincide with heat waves, interaction between air pollution and heat exposure can considerably increase impacts on cardiovascular and respiratory health (Shaposhnikov et al. 2014). This increase in impact is particularly important during severe heat waves, which are predicted to become more common as climate change proceeds (IPCC 2013).

Because fire is an inherent process in forest and grassland ecosystems in their natural state, wildfires and their emissions can be prevented only to a certain extent. However, vegetation management and other fire prevention and suppression practices are necessary near populated areas to reduce the risk of catastrophic events (Moreira et al. 2011; San-Miguel-Ayanz et al. 2013). In addition, emissions from agricultural-waste burning could be reduced by enacting more effective regulations on open-field fires, particularly in eastern European countries. Public health can also be protected by providing timely warnings and advice on how to reduce health risks during air pollution episodes; examples include staying indoors and keeping windows closed, avoiding physical activity in polluted outdoor environments, and using room air cleaners to improve indoor air quality (Elliott 2014). In particular, information and assistance should be targeted towards population groups most vulnerable to air pollution, namely the elderly, young children, and those with a preexisting respiratory or cardiovascular disease.

## Conclusions

Our findings suggest that exposure to PM_2.5_ originating from vegetation fires causes tens to hundreds of premature deaths each year in many European countries. The estimated impacts are highest in fire-prone regions in southern and eastern Europe, but other regions are also affected. Adverse effects of fire-originated air pollution on public health should be taken into consideration when evaluating the overall health and socioeconomic consequences of vegetation fires, which are expected to increase as climate change proceeds.

## Supplemental Material

(3.5 MB) PDF
