# Supplementary material for: Mortality due to Vegetation Fire–Originated PM2.5 Exposure in Europe—Assessment for the Years 2005 and 2008
Source: Environ Health Perspect. 2016 Jul 29;125(1):30–7. doi: 10.1289/EHP194 (PMC5226696; doi:10.1289/EHP194)
Supplement: (3.5 MB) PDF [file EHP194.s001.acco.pdf]

Environ Health Perspect
DOI: 10.1289/EHP194

Note to readers with disabilities: *EHP* strives to ensure that all journal content is accessible to all readers. However, some figures and Supplemental Material published in *EHP* articles may not conform to 508 standards due to the complexity of the information being presented. If you need assistance accessing journal content, please contact ehp508@niehs.nih.gov. Our staff will work with you to assess and meet your accessibility needs within 3 working days.

Supplemental Material

Mortality due to Vegetation-Fire Originated $PM_{2.5}$ Exposure in Europe – Assessment for the Years 2005 and 2008

Virpi Kollanus, Marje Prank, Alexandra Gens, Joana Soares, Julius Vira, Jaakko Kukkonen, Mikhail Sofiev, Raimo O. Salonen, and Timo Lanki

Table of Contents

Table S1. Relative frequency of the modelled daily average grid-cell concentrations of vegetation-fire originated $PM_{2.5}$ divided into seven exposure categories in 2005.

Table S2. Relative frequency of the modelled daily average grid-cell concentrations of vegetation-fire originated $PM_{2.5}$ divided into seven exposure categories in 2008.

Table S3. $PM_{2.5}$ emissions from vegetation fires in 2005 and 2008 (modelling domain extending from 35 to 70 degrees North and 15 degrees West to 35 degrees East).

Figure S1. Deviations of mean temperature (Celsius degrees) and total precipitation (mm) in 2005 and 2008 from the average in 2005-2011 (April-October). The temperature and precipitation data originate from the operational archives of the European Centre for Medium-Range Weather Forecasts (ECMWF 2016).

Figure S2. Mean wind speed (m/s, 500 m height from the ground) and direction in 2005 and 2008 (April-October). The wind data originate from the operational archives of the European Centre for Medium-Range Weather Forecasts (ECMWF 2016).

Figure S3. Annual variation in particulate matter (PM) emissions from vegetation fires in selected world regions based on the Integrated Monitoring System for Wildland Fires (IS4FIRES, FMI 2016a). Europe = the geographical Europe (17 degrees West to 50 degrees East, 33 to 75 degrees North).

Figure S4. Measured and modelled monthly average concentrations of $PM_{2.5}$ components at air-quality monitoring stations in a) Spain (strongly affected by vegetation fires) and b) Austria (mainly affected by other emission sources). Dots are the measured total $PM_{2.5}$ concentrations and shades are the stacked modelled concentrations of different components simulated by using the chemical transport model System for Integrated modeLling of Atmospheric coMposition (SILAM, FMI 2016b). PPMr is the primary anthropogenic $PM_{2.5}$, EC is elemental carbon, and fire-PM is vegetation-fire originated $PM_{2.5}$.

References

Table S1. Relative frequency of the modelled daily average grid-cell concentrations[a] of vegetation-fire originated $PM_{2.5}$ divided into seven exposure categories in 2005.

Region, country	<0.1 µg/m³	0.1-1 µg/m³	1-5 µg/m³	5-10 µg/m³	10-20 µg/m³	20-50 µg/m³	>50 µg/m³
Northern Europe	***91.6%***	***7.1%***	***1.2%***	***0.04%***	***0.01%***	***0.01%***	***0.001%***
Denmark	83.1%	14.6%	2.3%	0.01%	0%	0%	0%
Finland	92.0%	6.7%	1.2%	0.1%	0.03%	0.01%	0%
Norway	94.8%	4.6%	0.6%	0.02%	0.01%	0.01%	0%
Sweden	91.2%	7.3%	1.4%	0.03%	0.01%	0.004%	0.003%
Eastern Europe	***71.2%***	***20.4%***	***7.4%***	***0.5%***	***0.3%***	***0.2%***	***0.02%***
Bulgaria	64.5%	22.6%	10.0%	1.3%	1.0%	0.5%	0.1%
Czech Republic	68.7%	24.0%	6.8%	0.4%	0.1%	0.01%	0%
Estonia	88.0%	9.1%	2.8%	0.1%	0%	0%	0%
Hungary	66.2%	23.6%	9.1%	0.5%	0.3%	0.2%	0.02%
Latvia	84.3%	12.1%	3.5%	0.1%	0%	0%	0%
Lithuania	80.1%	15.1%	4.6%	0.1%	0.01%	0.02%	0.04%
Poland	71.7%	21.7%	6.4%	0.2%	0.03%	0.002%	0%
Romania	66.3%	21.4%	10.4%	0.7%	0.6%	0.5%	0.1%
Slovenia	74.4%	20.6%	4.5%	0.5%	0.1%	0.01%	0%
Slovakia	65.5%	24.8%	8.8%	0.6%	0.3%	0.1%	0%
Western Europe	***80.6%***	***15.8%***	***3.3%***	***0.2%***	***0.05%***	***0.005%***	***0.001%***
Austria	75.9%	18.9%	4.6%	0.5%	0.1%	0.01%	0%
Belgium	70.1%	21.4%	7.6%	0.8%	0.1%	0%	0%
France	78.8%	17.6%	3.2%	0.3%	0.1%	0.01%	0.003%
Germany	72.1%	22.6%	5.1%	0.2%	0.02%	0.001%	0%
Ireland	95.8%	3.8%	0.4%	0%	0%	0%	0%
Luxembourg	69.5%	21.7%	7.9%	0.5%	0.3%	0%	0%
Netherlands	70.3%	22.3%	6.9%	0.5%	0.05%	0%	0%
Switzerland	85.4%	13.0%	1.5%	0.1%	0%	0%	0%
United Kingdom	91.8%	6.7%	1.4%	0.1%	0.03%	0%	0%
Southern Europe	***72.2%***	***20.5%***	***5.4%***	***0.9%***	***0.5%***	***0.3%***	***0.2%***
Greece	64.7%	26.3%	7.8%	0.9%	0.2%	0.02%	0%
Italy	71.9%	22.3%	5.1%	0.6%	0.1%	0.03%	0%
Portugal	71.7%	15.3%	6.7%	2.0%	1.6%	1.5%	1.1%
Spain	76.9%	16.4%	4.2%	1.0%	0.7%	0.6%	0.2%
All regions	***78.6%***	***16.2%***	***4.3%***	***0.4%***	***0.2%***	***0.1%***	***0.1%***

[a]Estimated based on emissions from the Integrated Monitoring System for Wildland Fires (IS4FIRES, FMI 2016a) and chemical transport model the System for Integrated modeLling of Atmospheric coMposition (SILAM, FMI 2016b).

Table S2. Relative frequency of the modelled daily average grid-cell concentrations[a] of vegetation-fire originated $PM_{2.5}$ divided into seven exposure categories in 2008.

**Region, country**	**<0.1 µg/m³**	**0.1-1 µg/m³**	**1-5 µg/m³**	**5-10 µg/m³**	**10-20 µg/m³**	**20-50 µg/m³**	**>50 µg/m³**
Northern Europe	***94.6%***	***4.8%***	***0.5%***	***0.02%***	***0.0005%***	***0.001%***	***0%***
Denmark	89.2%	10.0%	0.8%	0.01%	0%	0%	0%
Finland	94.7%	4.5%	0.8%	0.02%	0%	0%	0%
Norway	97.0%	2.8%	0.1%	0.005%	0%	0.003%	0%
Sweden	94.5%	5.0%	0.5%	0.02%	0.001%	0%	0%
Eastern Europe	***72.0%***	***20.3%***	***5.8%***	***1.0%***	***0.7%***	***0.3%***	***0.01%***
Bulgaria	58.7%	23.8%	11.7%	2.8%	2.0%	1%	0.04%
Czech Republic	73.1%	23.3%	3.4%	0.2%	0.01%	0%	0%
Estonia	86.9%	10.3%	2.6%	0.1%	0.1%	0.0%	0%
Hungary	65.1%	25.6%	8.1%	0.8%	0.4%	0.1%	0%
Latvia	84.6%	12.3%	2.7%	0.2%	0.1%	0.1%	0%
Lithuania	82.9%	13.4%	3.4%	0.2%	0.04%	0.1%	0%
Poland	75.8%	20.9%	3.1%	0.1%	0.1%	0.005%	0%
Romania	65.9%	20.8%	8.3%	2.2%	1.7%	1.0%	0.1%
Slovenia	75.7%	20.2%	4.1%	0.1%	0.04%	0%	0%
Slovakia	67.7%	25.4%	6.3%	0.4%	0.2%	0%	0%
Western Europe	***85.0%***	***12.9%***	***2.0%***	***0.1%***	***0.02%***	***0.001%***	***0%***
Austria	81.2%	15.3%	3.0%	0.4%	0.1%	0.01%	0%
Belgium	77.4%	17.7%	4.8%	0.1%	0%	0%	0%
France	86.4%	11.6%	1.9%	0.1%	0.02%	0.001%	0%
Germany	78.3%	18.9%	2.8%	0.1%	0.02%	0%	0%
Ireland	93.6%	5.9%	0.5%	0.03%	0%	0%	0%
Luxembourg	82.0%	14.7%	3.2%	0.1%	0%	0%	0%
Netherlands	74.8%	20.4%	4.6%	0.1%	0.02%	0%	0%
Switzerland	90.3%	9.0%	0.7%	0.04%	0%	0%	0%
United Kingdom	90.5%	8.4%	1.1%	0.01%	0%	0%	0%
Southern Europe	***76.6%***	***16.8%***	***5.4%***	***0.9%***	***0.3%***	***0.05%***	***0.002%***
Greece	61.7%	22.7%	11.8%	2.7%	1.0%	0.1%	0%
Italy	71.3%	20.9%	6.9%	0.7%	0.2%	0.1%	0.01%
Portugal	88.8%	10.2%	0.9%	0.1%	0.01%	0%	0%
Spain	87.6%	11.1%	1.2%	0.1%	0.02%	0.01%	0%
All regions	***82.0%***	***13.7%***	***3.5%***	***0.5%***	***0.2%***	***0.1%***	***0.004%***

[a]Estimated based on emissions from the Integrated Monitoring System for Wildland Fires (IS4FIRES, FMI 2016a) and chemical transport model the System for Integrated modeLling of Atmospheric coMposition (SILAM, FMI 2016b).

Table S3. $PM_{2.5}$ emissions[a] from vegetation fires in 2005 and 2008 (modelling domain extending from 35 to 70 degrees North and 15 degrees West to 35 degrees East).

Country	2005 [Ton]	2008 [Ton]
Algeria	64044	54728
Morocco	9064	696
Tunisia	3751	6342
Albania	2529	6919
Andorra	9	0
Austria	11900	14081
Belarus	4200	6111
Belgium	1557	1085
Bosnia and Herzegovina	816	5907
Bulgaria	22526	28917
Croatia	2206	4752
Czech	2791	2326
Denmark	987	426
Estonia	68	3752
Finland	6820	1177
France	47656	23931
Germany	25652	25649
Greece	13978	40246
Hungary	6186	9937
Ireland	188	510
Italy	153542	227692
Latvia	338	52
Lithuania	44333	329
Luxemburg	273	128
Macedonia	18660	23290
Malta	0	0
Moldova	6665	19194
Netherlands	9244	8344
Norway	1626	9264
Poland	11123	12448
Portugal	591463	9467
Romania	52407	111064
Russia	20479	34247
Serbia & Montenegro	5250	20374
Slovakia	5291	3886
Slovenia	116	71
Spain	262075	30464
Sweden	5883	6419
Switzerland	22	61
Turkey	30040	57922
Ukraine	179094	390773
United Kingdom	7243	8492
**TOTAL**	**1632096**	**1211473**

[a]Estimated based on emissions from the Integrated Monitoring System for Wildland Fires (IS4FIRES, FMI 2016a)

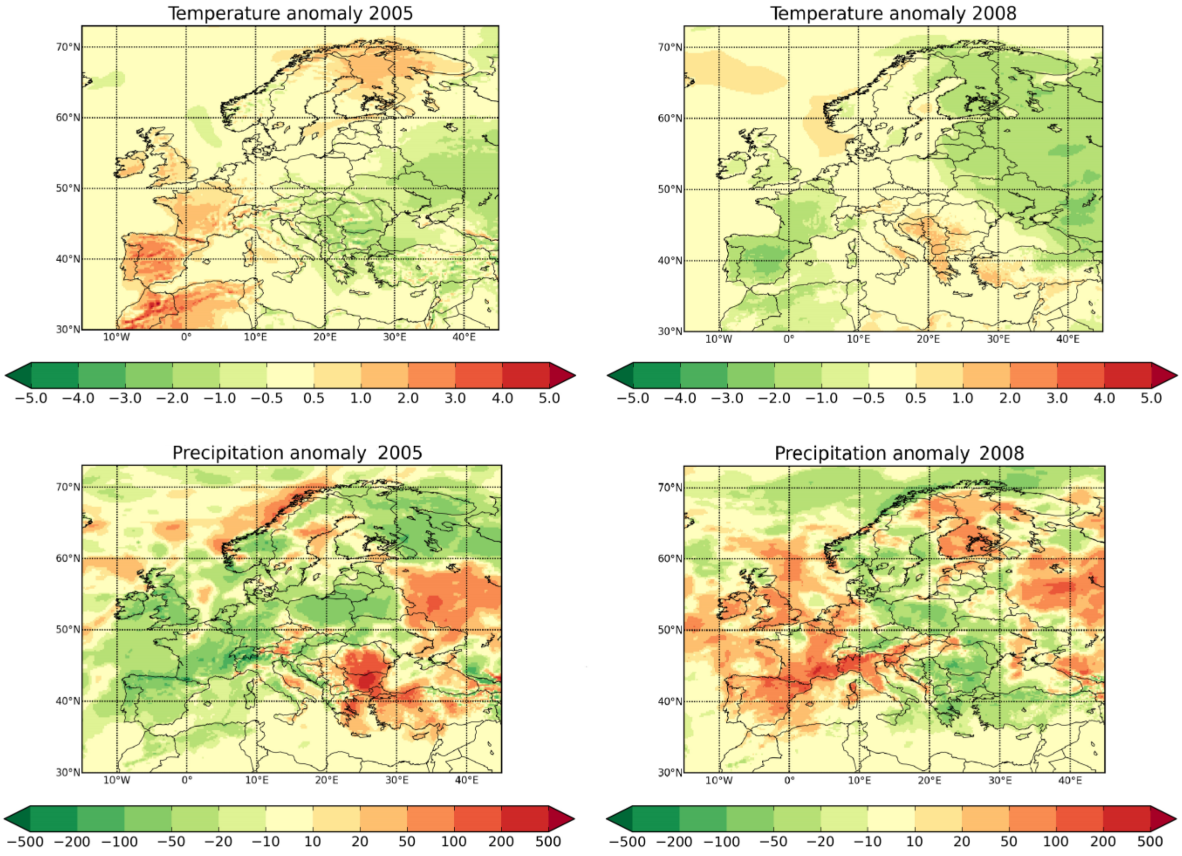

Figure S1. Deviations of mean temperature (Celsius degrees) and total precipitation (mm) in 2005 and 2008 from the average in 2005-2011 (April-October). The temperature and precipitation data originate from the operational archives of the European Centre for Medium-Range Weather Forecasts (ECMWF 2016).

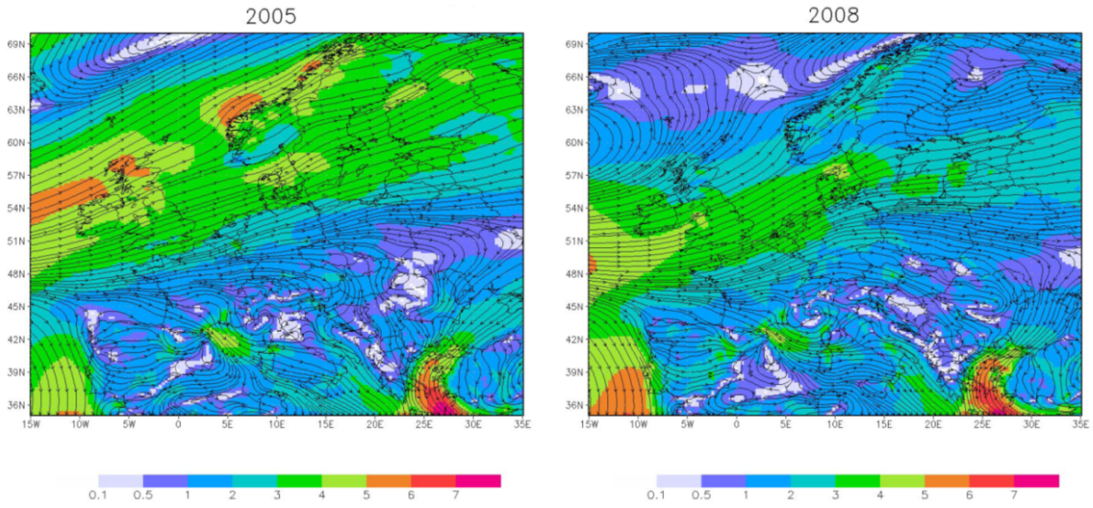

Figure S2. Mean wind speed (m/s, 500 m height from the ground) and direction in 2005 and 2008 (April-October). The wind data originate from the operational archives of the European Centre for Medium-Range Weather Forecasts (ECMWF 2016).

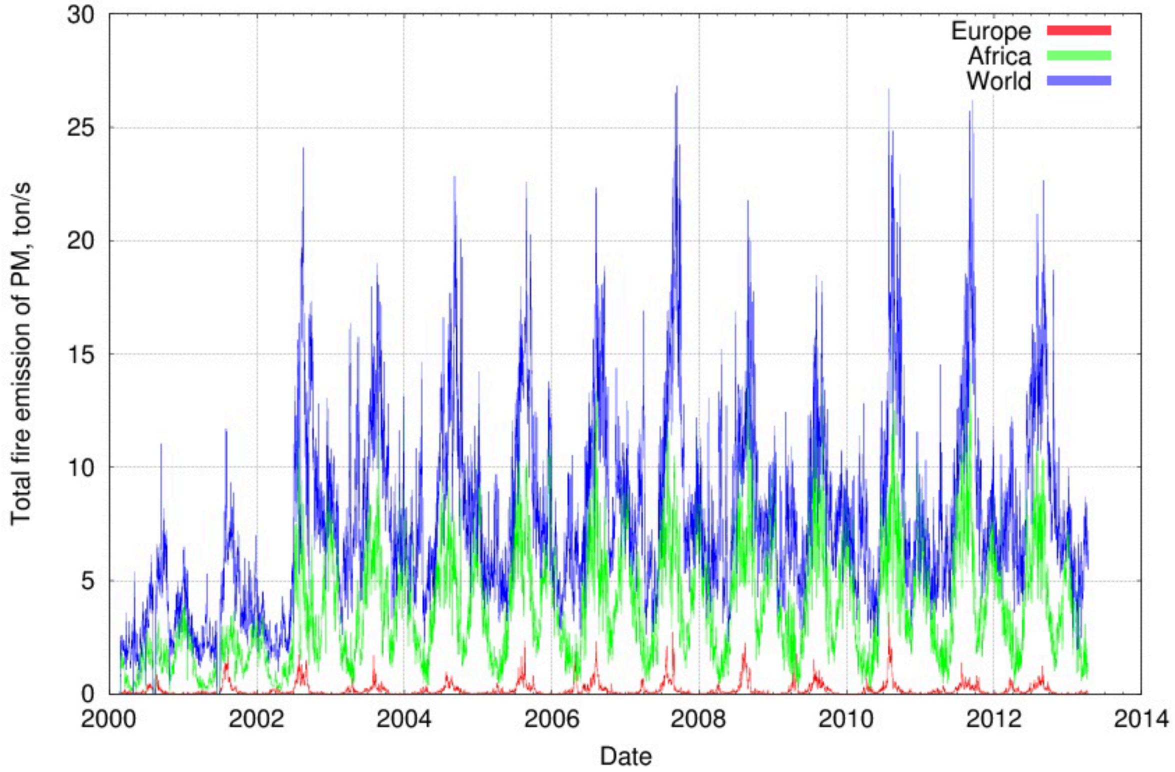

Figure S3. Annual variation in particulate matter (PM) emissions from vegetation fires in selected world regions based on the Integrated Monitoring System for Wildland Fires (IS4FIRES, FMI 2016a). Europe = the geographical Europe (17 degrees West to 50 degrees East, 33 to 75 degrees North).

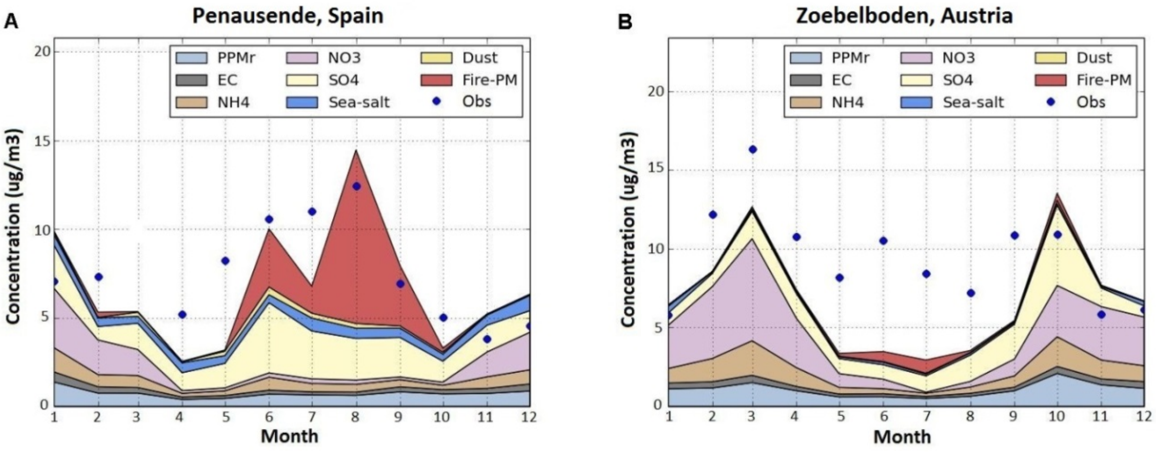

Figure S4. Measured and modelled monthly average concentrations of $PM_{2.5}$ components at air-quality monitoring stations in a) Spain (strongly affected by vegetation fires) and b) Austria (mainly affected by other emission sources). Dots are the measured total $PM_{2.5}$ concentrations and shades are the stacked modelled concentrations of different components simulated by using the chemical transport model System for Integrated modeLling of Atmospheric coMposition (SILAM, FMI 2016b). PPMr is the primary anthropogenic $PM_{2.5}$, EC is elemental carbon, and fire-PM is vegetation-fire originated $PM_{2.5}$.

References

ECMWF (European Centre for Medium-Range Weather Forecasts). 2016. Set I - Atmospheric Model high resolution 10-day forecast (HRES). Available: http://www.ecmwf.int/en/forecasts/datasets/set-i [accessed 18 May 2016].

FMI (Finnish Meteorological Institute). 2016a. IS4FIRES – An integrated monitoring and modelling system for wildland fires. Available: http://is4fires.fmi.fi/ [accessed 27 May 2016].

FMI (Finnish Meteorological Institute). 2016b. SILAM v.5.x. Available: http://silam.fmi.fi/ [accessed 27 May 2016].
